# Intramolecular C–H arylation of pyridine derivatives with a palladium catalyst for the synthesis of multiply fused heteroaromatic compounds

**DOI:** 10.3762/bjoc.20.269

**Published:** 2024-12-13

**Authors:** Yuki Nakanishi, Shoichi Sugita, Kentaro Okano, Atsunori Mori

**Affiliations:** 1 Department of Chemical Science and Engineering, Kobe University, 1-1 Rokkodai, Nada, Kobe 657-8501, Japan; 2 Research Center for Membrane and Film Technology, Kobe University, 1-1 Rokkodai, Nada, Kobe 657-8501, Japan

**Keywords:** intramolecular C–H arylation, multiply fused heterocycles, palladium acetate, phosphine ligand, pyridine amides

## Abstract

The C–H arylation of 2-quinolinecarboxyamide bearing a C–Br bond at the *N*-aryl moiety is carried out with a palladium catalyst. The reaction proceeds at the C–H bond on the pyridine ring adjacent to the amide group in the presence of 10 mol % Pd(OAc)_2_ at 110 °C to afford the cyclized product in 42% yield. The yield is improved to 94% when the reaction is performed with PPh_3_ as a ligand of palladium. The reaction is examined with amides derived from unsubstituted picoline, 6-methylpicoline, and 2,6-pyridinedicarboxylic acid in a similar manner to afford the cyclized products in 70%, 77%, and 87% yield, respectively. The related reaction is also carried out with amides of non-pyridine derivatives terephthal- and benzamides to afford multiply fused heterocyclic compounds in 81% and 89% yields, respectively.

## Introduction

**Figure 1:**
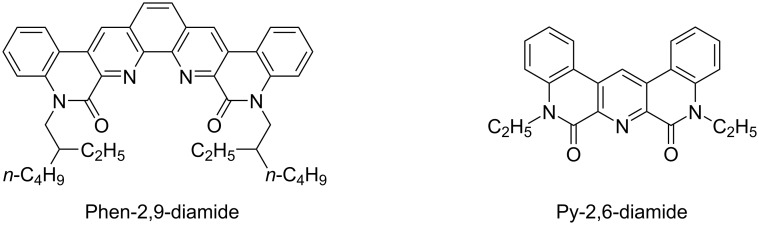
Structures of multiply fused heterocyclic compounds composed of pyridine rings.

Transition-metal-catalyzed synthetic reactions have recently attracted much attention in synthetic organic chemistry [1–2]. C–H Arylation reactions catalyzed by a transition metal are of particular interest because these reactions involve rather superior efficiencies in atom economy [3–4]. The extension of the reaction to an intramolecular version represents a viable approach for the construction of several fused-ring skeletons [5]. Such ring structures containing heterocyclic rings would be of crucial importance because heterocycle-fused ring structures [6–7] are found in a variety of advanced materials [8–9] and biologically important molecules [10–12]. A wide range of pyridine derivatives have been employed as extractants of metal ions through chelation [13]. Phenanthrolines, a class of pyridine derivatives, have attracted attention for the efficient and selective extraction of lanthanides and actinides and, furthermore, a number of heterocycles involving pyridine rings have been reported to exhibit biological activities [14–22]. We have recently reported, as shown in Figure 1, that the introduction of a multiply fused structure toward a phenanthroline diamide (Phen-2,9-diamide) [23] can be achieved by employing a palladium-catalyzed intramolecular C–H arylation [24–28]. One of the thus obtained products exhibited a remarkable extraction performance for a lanthanide ion, in which a metal-specific extraction was found despite the similarities in the lanthanide series [23]. Chakravorty and co-workers reported that a similar arylation reaction gave access to the fused skeleton of the diamide of 2,6-pyridinedicarboxlic acid (Py-2,6-diamide) [29]. Our interest has thus turned to extend the substrate scope of the palladium-catalyzed C–H arylation of phenanthroline to other nitrogen-containing heteroaromatic compounds. It is therefore intriguing to demonstrate the advantage of the palladium-catalyzed intramolecular C–H arylation compared to other protocols for the construction of related ring structures [30–32]. We herein report the palladium-catalyzed intramolecular C–H arylation of several pyridine and non-pyridine amides to afford multiply fused heterocyclic compounds.

## Results and Discussion

**Scheme 1:**
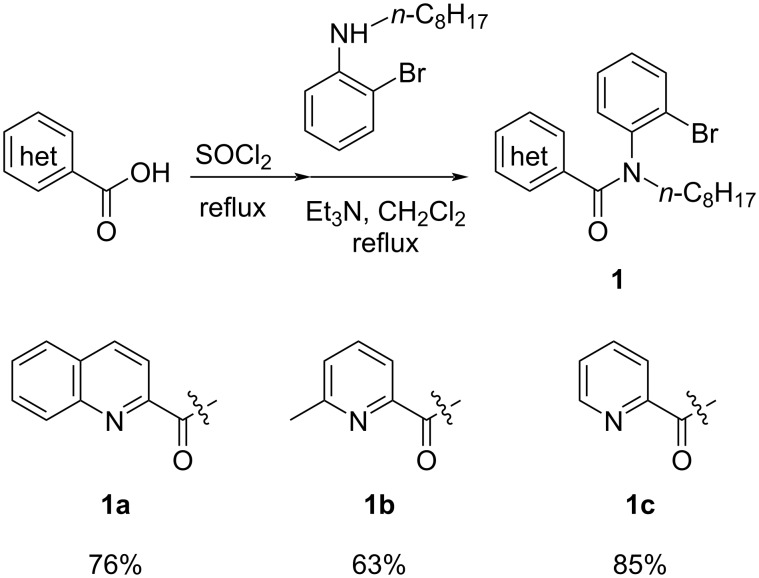
Synthesis of C–H arylation precursors **1a**–**c**.

First, we started with the synthesis of the cyclization precursors **1a**–**c** that was carried out by the reaction of the corresponding heteroaromatic carboxylic acids with thionyl chloride followed by treatment with *N*-octyl-2-bromoaniline [15]. The reactions proceeded smoothly affording products **1a**–**c** in good yields as shown in Scheme 1.

We then studied the reaction of quinoline amide **1a** under several conditions. We carried out the palladium-catalyzed intramolecular coupling reaction of precursor **1a** under similar conditions [23], which afforded smooth reaction with phenanthroline bisamide, with 10 mol % of palladium acetate as a catalyst in the presence of potassium carbonate and tetra-*n*-butylammonium bromide in *N*,*N*-dimethylacetamide (DMA). Table 1 summarizes the results. The yield of the reaction improved as the temperature was increased from 90 °C to 130 °C (Table 1, entries 1–3). When the reaction was carried out at 150 °C, the yield decreased to 27%. A longer reaction period of 72 h at 130 °C also resulted in a decreased yield (27%) (Table 1, entries 4 and 5). It was found that increasing the amount of potassium carbonate to a three-fold excess improved the yield of **2a** to 59% in the reaction at 110 °C shown in entry 6 of Table 1. Next, the effect of the ligand of the palladium catalyst was examined. The addition of ligand improved the yield of **2a** as shown in Table 1, entries 7–10. Among several ligands, including Buchwald-type phosphines **L1**–**L4** [33] examined, it was found that the use CyJohnPhos (**L3**) afforded the cyclized product in 90% yield and the reaction with PPh_3_ (**L4**) as a ligand was also effective to afford **2a** in 94% yield.

**Table 1:** Studies on the reaction conditions for **2a** from **1a**.

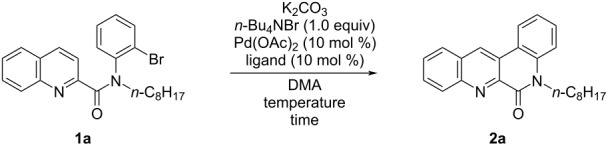					

Entry	Temp. (°C)	K_2_CO_3_ (equiv)	Time (h)	Ligand	Yield (%)^a^

1	90	1.0	24	none	7^b^
2	110	1.0	24	none	42
3	130	1.0	24	none	49
4	150	1.0	24	none	27
5	130	1.0	72	none	27^b^
6	110	3.0	24	none	59
7	110	3.0	24	**L1** ^c^	58
8	110	3.0	24	**L2** ^d^	69
9	110	3.0	24	**L3** ^e^	90
10	110	3.0	24	**L4** ^f^	94 (87^b^)

^a^Yield determined by ^1^H NMR with 1,1,2,2-tetrachloroethane as an internal standard; ^b^isolated yield; ^c^**L1**: SPhos = 2-dicyclohexylphosphino-2’,6’-dimethoxybiphenyl; ^d^**L2**: PCy_3_ = tricyclohexylphosphine; ^e^**L3**: CyJohnPhos = 2-(dicyclohexylphosphino)biphenyl; ^f^**L4**: PPh_3_ = triphenylphosphine.

The reaction was then carried out with several pyridine derivatives including amide derivatives composed of 6-methylpicoline (**1b**) and unsubstituted picoline (**1c**) as summarized in Table 2. When the reaction was examined in the absence of a phosphine ligand, the yields of the cyclized products **2b** and **2c** were much worse compared to the same reaction of **1a**. In the latter case product **2a** was obtained in 59% yield, whereas the yields for **2b** and **2c** were only 18% and 5%, respectively. The use of PPh_3_ (**L4**) as a ligand slightly improved the yields of **2b** and **2c** to 58% and 24%, respectively. The highest yield of **2b** was obtained in the presence of CyJohnPhos (**L3**) as ligand, while tricyclohexylphosphine (**L2**) gave the best yield in the reaction of **1c**. Concerning the reaction of **1c**, the use of tetra-*n*-butylammonium bromide and pivalic acid as additives and PCy_3_ (**L2**) as a ligand further improved the yield to 77%. Chakravorty and co-workers showed that a smooth reaction proceeded with pyridine 2,6-dicarboxylic acid bisamide **3** [29] and we thus compared the reaction of **3** under similar conditions to that of **1a**. The reaction afforded product **4** in 87% yield, which was found to be comparable with the case of **1a**. The reactivity toward the palladium-catalyzed cyclization was thus shown as **3** ≈ **1a** >> **1b** > **1c**. The related trend was also observed in the reaction of phenanthroline monoamide **5a** and diamide **5b**. The reaction of **5a** afforded the cyclized product in 51% yield, which contrasted with our previous result for the cyclization of **5b** to afford the doubly cyclized product **6b** (reported yield: 85% [23]), suggesting that the superior reactivity was found for bifunctional bisamides compared to monoamides.

**Table 2:** Pd-catalyzed C–H arylation of heteroarenes.

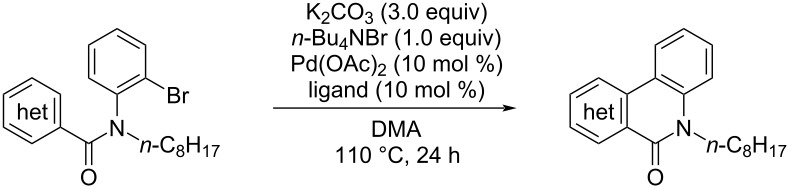				

Substrate	Conc.^a^	Ligand	Product	Yield^b^

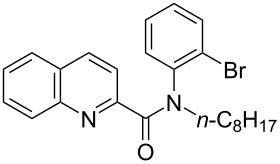 **1a**	0.033	none	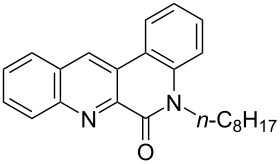 **2a**	59%
**1a**	0.033	**L4**	**2a**	94% (87%)
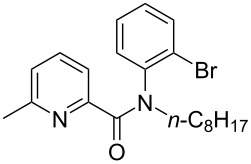 **1b**	0.033	none	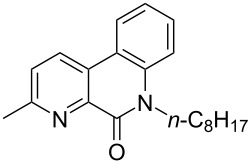 **2b**	18%
**1b**	0.033	**L2**	**2b**	47%
**1b**	0.033	**L4**	**2b**	58%
**1b**	0.033	**L3**	**2b**	70% (52%)
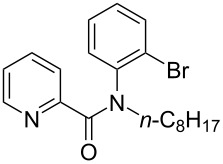 **1c**	0.033	none	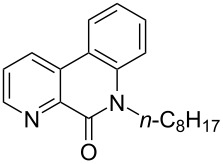 **2c**	(5%)
**1c**	0.067	none	**2c**	11%
**1c**	0.067	**L4**	**2c**	24%
**1c**	0.067	**L3**	**2c**	33%
**1c**	0.067	**L2**	**2c**	42%
**1c**	0.067	**L2**	**2c**	77%^c^ (62%)
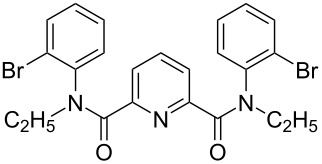 **3**	0.032	none	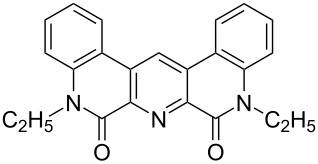 **4**	87%^d^
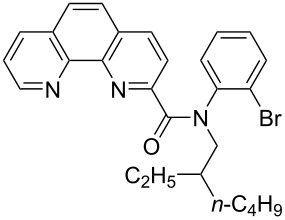 **5a**	0.032	none	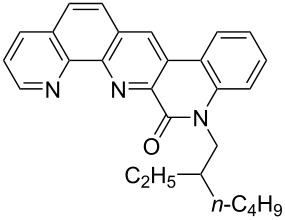 **6a**	(51%)
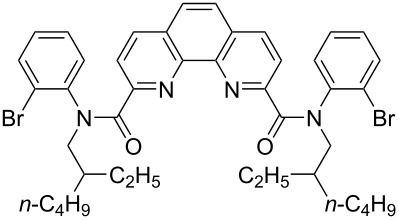 **5b**	0.032	none	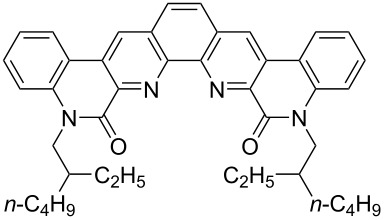 **6b**	85%^d,e^

^a^Substrate/DMA (mol/L); ^b^yield determined by ^1^H NMR with 1,1,2,2-tetrachloroethane as an internal standard and isolated yield is given in parenthesis; ^c^*n*-Bu_4_NBr/*t*-BuCOOH 1:1 was used as an additive; ^d^2.0 equiv of *n*-Bu_4_NBr was used; ^e^result taken from [23].

**Scheme 2:**
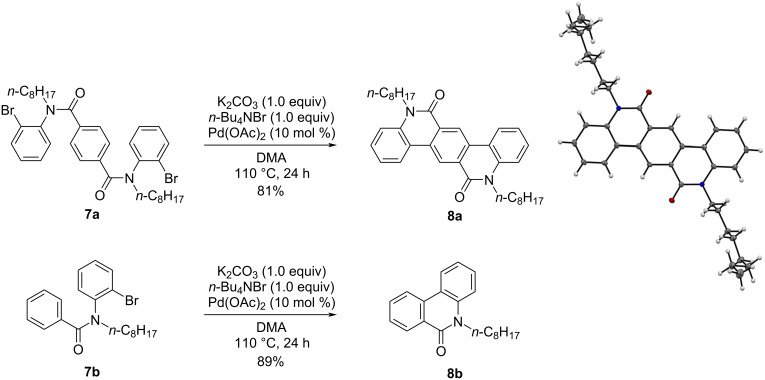
Palladium-catalyzed intramolecular direct arylation for synthesizing **8a** and **8b** and the X-ray crystallographic structure of **8a**.

It was also found that the reaction also is applicable to a carbocyclic amide derivative. When the reaction was carried out with **7a** under similar conditions, the cyclization occurred to afford **8a** in 81% yield as shown in Scheme 2. The formation of **8a** was confirmed by X-ray crystallographic analysis (CCDC 2227450). The related monofunctionalized analog **7b** also smoothly underwent cyclization to afford **8b** in 89% yield under similar conditions, in which the result of carbocyclic amide (**7a** vs **7b**) contrasted with the case of heterocyclic ones, **1c** vs **3** and **5a** vs **5b**.

## Conclusion

We have shown the facile synthesis of fused nitrogen-containing heterocycles and extended the scope of the intramolecular palladium catalyzed C–H arylation to pyridine derivatives. The cyclization reaction proceeded in a moderate to excellent yield when an appropriate phosphine ligand was employed. The reaction is expected to be useful for the synthesis of functional materials, and bioactive molecules in a facile manner.

## Experimental

**Typical experimental procedure for the C–H arylation of pyridine derivative 5-octyldibenzo[*b*,*f*][1,7]naphthyridin-6(5*H*)-one (2a):** To a screw-capped test tube equipped with a magnetic stirring bar were added amide **1a** (44.1 mg, 0.100 mmol), potassium carbonate (42.0 mg, 0.304 mmol), tetrabutylammonium bromide (31.7 mg, 0.098 mmol), Pd(OAc)_2_ (2.2 mg, 10 mol %), and triphenylphosphine (2.8 mg, 10 mol %). The mixture was dissolved in 3.1 mL of DMA and stirring was continued at 110 °C for 24 h. Then, water (3 mL) was added after cooling to room temperature. The product was extracted with dichloromethane (2 mL) three times. The combined organic extracts were repeatedly washed with water (20 mL) and brine (20 mL). The organic layer was dried over anhydrous sodium sulfate and concentrated under reduced pressure to give a crude material, which was purified by silica gel column chromatography (hexane/MeOAc 1:1) to give 31.0 mg (87% yield) of **2a** as a colorless solid. (NMR yield: 94%); mp 85.1–86.6 °C; ^1^H NMR (CDCl_3_) δ 8.98 (s, 1H), 8.43 (d, *J* = 8.4 Hz, 1H), 8.31 (dd, *J* = 8.0, 1.2 Hz, 1H), 7.95 (d, *J* = 8.4 Hz, 1H), 7.76 (ddd, *J* = 8.0, 7.6, 1.2 Hz, 1H), 7.62 (ddd, *J* = 7.6, 7.6, 1.2 Hz, 1H), 7.54 (ddd, *J* = 8.4, 8.0, 1.2 Hz, 1H), 7.37 (d, *J* = 8.4 Hz, 1H), 7.30 (dd, *J* = 8.0, 7.6 Hz, 1H), 4.40 (dd, *J* = 8.0, 7.6 Hz, 2H), 1.76–1.88 (m, 2H), 1.44–1.56 (m, 2H), 1.18–1.42 (m, 8H), 0.86 (t, *J* = 6.8 Hz, 3H); ^13^C{^1^H} NMR (CDCl_3_) δ 160.1, 148.4, 142.0, 136.7, 131.1, 130.5, 130.2, 130.1, 129.1, 128.7, 127.7, 126.3, 123.8, 122.7, 118.3, 115.4, 43.3, 31.9, 29.5, 29.3, 27.3, 27.1, 22.7, 14.2; IR (ATR): 2959, 2929, 2856, 1661, 751 cm^−1^; HRMS–DART^+^ (*m*/*z*): [M + H]^+^ calcd for C_24_H_27_N_2_O, 359.2123; found, 359.2134.

## Supporting Information

Accession code CCDC 2227450 contains the supplementary crystallographic data for **8a**. This data can be obtained free of charge via https://www.ccdc.cam.ac.uk/structures, or by emailing data_request@ccdc.cam.ac.uk, or by contacting Cambridge Crystallographic Data Centre, 12 Union Road, Cambridge CB2 1EZ, UK; fax: +44 1223 336033.

File 1 Additional experimental details and copies of ^1^H and ^13^C{^1^H} NMR spectra.

## Data Availability

Additional research data generated and analyzed during this study is not shared.
