# Supplementary material for: Intramolecular C–H arylation of pyridine derivatives with a palladium catalyst for the synthesis of multiply fused heteroaromatic compounds
Source: Beilstein J Org Chem. 2024 Dec 13;20:3256–62. doi: 10.3762/bjoc.20.269 (PMC11650519; doi:10.3762/bjoc.20.269)
Supplement: File 1 — Additional experimental details and copies of 1H and 13C{1H} NMR spectra. [file Beilstein_J_Org_Chem-20-3256-s001.pdf]

BEILSTEIN JOURNAL OF ORGANIC CHEMISTRY

Supporting Information

for

Intramolecular C–H arylation of pyridine derivatives with a palladium catalyst for the synthesis of multiply fused heteroaromatic compounds

Yuki Nakanishi, Shoichi Sugita, Kentaro Okano and Atsunori Mori

Beilstein J. Org. Chem. **2024,** *20,* 3256–3262. doi:10.3762/bjoc.20.269

Additional experimental details and copies of ^{1}H and ^{13}C{^{1}H} NMR spectra

License and Terms: This is a supporting information file under the terms of the Creative Commons Attribution License (https://creativecommons.org/licenses/by/4.0). Please note that the reuse, redistribution and reproduction in particular requires that the author(s) and source are credited and that individual graphics may be subject to special legal provisions.
The license is subject to the *Beilstein Journal of Organic Chemistry* terms and conditions: (https://www.beilstein-journals.org/bjoc/terms)

Table of contents

Experimental section.. S2

Copies of NMR spectra.. S8

References.. S18

Experimental

General. Analytical thin-layer chromatography (TLC) was performed on Merck 60 F_{254} aluminum sheets precoated with a 0.25 mm thickness of silica gel. Flash column chromatography was performed on Wakogel® C-300 (45–75 μm, Fujifilm Wako Pure Chemical Co.). Melting points (mp) were measured on a Yanaco MP-J3 and are uncorrected. Infrared (IR) spectra were recorded on a Bruker Alpha with an ATR attachment (Ge) and are reported in wavenumbers (cm^{-1}). ^{1}H NMR (400 MHz) and $^{13}C\{^{1}H\}$ NMR (100 MHz) spectra were measured on a JEOL ECZ400 spectrometer. Chemical shifts for ^{1}H NMR are reported in parts per million (ppm) downfield from tetramethylsilane with the solvent resonance as internal standards ($CHCl_3$: δ 7.26 ppm, tetramethylsilane: δ 0 ppm). The following abbreviations are used for spin multiplicity: s = singlet, d = doublet, t = triplet, quint = quintet, and m = multiplet. Chemical shifts for $^{13}C\{^{1}H\}$ NMR are reported in ppm from tetramethylsilane with the solvent resonance as the internal standard ($CDCl_3$: δ 77.16 ppm). The measurement of high-resolution mass spectrum (HRMS) was performed on a JEOL JMS-T100LP AccuTOF LC-Plus (ESI) with a JEOL MS-5414DART attachment.

Materials. *N*,*N*-Dimethylacetamide (DMA) and dichloromethane were purchased from Fujifilm Wako Pure Chemical Co. as an anhydrous grade. Preparation of *N*-octyl-2-bromoaniline [1], pyridine diamide **3** [2], and phenanthroline monoamide **5a** [1] was carried out in a manner described in the literature. Other chemicals were purchased and used as received without further purification.

***N*-(2-Bromophenyl)-*N*-octyl-2-quinolinecarboxamide (1a)**

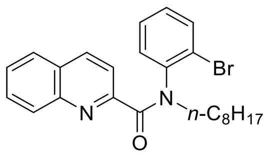

The preparation of **1** was carried out in a manner described in the literature [3]. To a flame-dried 200 mL round-bottomed flask equipped with a Teflon-coated magnetic stirring bar, a three-way stopcock, and a rubber septum were added 2-quinolinecaboxylic acid (797.6 mg, 4.61 mmol) and 21.2 mL of thionyl chloride under nitrogen atmosphere. After stirring at 80 °C for 19 h, thionyl chloride was removed under reduced pressure and the residue was dissolved in dichloromethane. To the solution were added *N*-octyl-2-bromoaniline (1.40 g, 4.9 mmol) and triethylamine (5.1 mL) and the solution was stirred at 40 °C for 5 h. The reaction mixture was poured into 37% hydrochloric acid (12 mL) to

result in phase separation. The aqueous layer was extracted with dichloromethane (10 mL) three times. The combined organic extracts were washed with brine (60 mL), dried over anhydrous sodium sulfate. The solution was concentrated under reduced pressure to leave a crude material, which was purified by silica gel column chromatography (hexane/MeOAc 2:1) to provide 1.53 g (76% yield) of **1a** as a colorless solid. Mp 54.3–56.0 ºC; ^{1}H NMR ($CDCl_3$): δ 8.05 (d, *J* = 8.8 Hz, 1H), 7.78 (d, *J* = 8.8 Hz, 1H), 7.69 (dd, *J* = 8.8, 8.8 Hz, 2H), 7.58 (ddd, *J* = 7.2, 7.2, 1.6 Hz, 1H), 7.47 (ddd, *J* =7.2, 7.2, 1.6 Hz, 1H), 7.43 (dd, *J* =7.6, 1.2 Hz, 1H), 7.35 (dd, *J* = 8.0, 2.0 Hz, 1H), 7.18 (ddd, *J* = 8.0, 7.6, 1.2 Hz, 1H), 7.01 (ddd, *J* = 7.6, 7.2, 1.2 Hz, 1H), 4.20–4.30 (m, 1H), 3.52–3.61 (m, 1H), 1.60–1.84 (m, 2H), 1.20–1.48 (m, 10H), 0.87 (t, *J* = 7.2 Hz, 3H) and 5–10% amide-derived rotational isomer signals; ^{13}C{^{1}H} NMR ($CDCl_3$): δ 168.0, 153.4, 146.3, 142.2, 136.3, 133.2, 131.8, 129.8, 129.6, 129.4, 128.7, 127.81, 127.75, 127.49, 127.47, 123.5, 120.6, 49.9, 31.9, 29.5, 29.4, 27.4, 27.2, 22.8, 14.3, and amide-derived rotational isomer signals (168.5, 154.0, 146.8, 140.8, 137.1, 133.8, 130.6, 130.1, 130.0, 129.4, 128.6, 127.7, 123.3, 121.0, 52.2, 31.8, 29.3, 28.9, 26.7, 22.7); IR (ATR): 2926, 2855, 1651, 1475, 774, 761 cm^{-1}; HRMS (DART+) m/z calcd. for $C_{24}H_{28}{}^{79}BrN_2O$, 439.1385 [M+H]$^+$; found, 439.1382.

Synthesis of **1b**, **1c**, **7a**, and **7b** was carried out in a similar manner, spectroscopic characteristics and analytical properties are shown below.

***N*-(2-Bromophenyl)-*N*-octyl-6-methylpicolinamide (1b)**

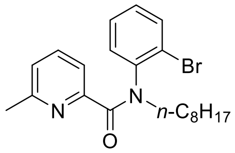

Isolated yield: 63%; ^{1}H NMR ($CDCl_3$): δ 7.36–7.49 (m, 3H), 7.13–7.24 (m, 2H), 7.04 (ddd, *J* = 8.0, 7.2, 2.0 Hz, 1H), 6.92 (dd, *J* =7.2, 1.2 Hz, 1H), 4.14–4.23 (m, 1H), 3.42–3.51 (m, 1H), 2.22 (s, 3H), 1.51–1.81 (m, 2H), 1.07–1.44 (m, 10H), 0.86 (t, *J* = 7.2 Hz, 3H) and 5–10% amide-derived rotational isomer signals; ^{13}C{^{1}H} NMR ($CDCl_3$): δ 168.1, 156.7, 153.0, 142.2, 136.2, 133.1, 131.9, 128.6, 127.5, 123.6, 123.5, 120.4, 49.5, 31.8, 29.4, 29.3, 27.3, 27.1, 23.9, 22.7, 14.1, and amide-derived rotational isomer signals (168.5, 157.4, 137.0, 133.7, 130.6, 129.2, 128.4, 124.2, 123.3, 120.6, 53.5, 52.0, 28.7, 26.7, 24.5); IR (ATR): 2926, 2856, 1653, 1587, 1476, 753 cm^{-1}; HRMS (DART+) m/z calcd. for $C_{21}H_{28}{}^{79}BrN_2O$, 403.1385 [M+H]$^+$; found, 403.1389.

***N*-(2-Bromophenyl)-*N*-octylpicolinamide (1c)**

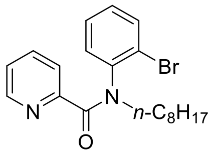

Isolated yield: 85%; ^{1}H NMR ($CDCl_3$): δ 8.20–8.25 (m, 1H), 7.52–7.58 (m, 2H), 7.43 (dd, *J* = 8.0, 1.6 Hz, 1H), 7.23 (dd, *J* = 8.0, 1.6 Hz, 2H), 7.16 (ddd, *J* = 8.0, 7.2, 1.6 Hz, 2H), 4.16–4.25 (m, 1H), 3.41–3.49 (m, 1H), 1.52–1.78 (m, 2H), 1.07–1.42 (m, 10H), 0.80–0.91 (m, 3H) and 5–10% amide-derived rotational isomer signals; $^{13}C\{^{1}H\}$ NMR ($CDCl_3$): δ 168.3, 154.2, 148.0, 141.8, 136.1, 133.3, 132.0, 128.9, 127.8, 124.0, 123.4, 123.3, 49.5, 31.9, 29.4, 29.3, 27.4, 27.1, 22.7, 14.2, and amide derived rotational isomer signals (168.4, 154.6, 148.5, 140.6, 137.0, 133.8, 130.6, 129.3, 128.5, 124.7, 123.8, 51.9, 31.8, 29.2, 29.1, 28.7, 26.6); IR (ATR): 2927, 2855, 1654, 1585, 746 cm^{-1}; HRMS (DART+) m/z calcd. for $C_{20}H_{26}{}^{79}BrN_2O$, 389.1229 $[M+H]^+$; found, 389.1214.

***N*^{1},*N*^{4}-Bis(2-bromophenyl)-*N*^{1},*N*^{4}-dioctylterephthalamide (7a)**

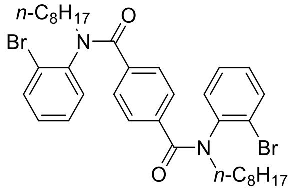

Isolated yield: 81%; Mp 133.6–134.6 ºC; ^{1}H NMR ($CDCl_3$): δ 7.46 (d, *J* = 8.0 Hz, 2H), 6.99–7.14 (m, 8H), 6.95 (ddd, *J* = 8.0, 7.6, 1.2 Hz, 2H), 4.07–4.18 (m, 2H), 3.30–3.40 (m, 2H), 1.57–1.67 (m, 2H), 1.41–1.53 (m, 2H), 1.13–1.35 (m, 20H), 0.86 (t, *J* = 6.8 Hz, 6H); $^{13}C\{^{1}H\}$ NMR ($CDCl_3$): δ 169.9, 141.7, 137.2, 133.9, 131.8, 131.7, 129.2, 128.2, 127.3, 123.4, 123.3, 49.4, 49.3, 31.9, 29.4, 29.3, 27.3, 27.1, 22.7, 14.2; IR (ATR): 2956, 2926, 2857, 1638, 731 cm^{-1}; HRMS (DART+) m/z calcd. for $C_{36}H_{47}{}^{79}Br^{81}BrN_2O_2$, 699.1984 $[M+H]^+$; found, 699.1955.

***N*-(2-Bromophenyl)-*N*-octylbenzamide (7b)**

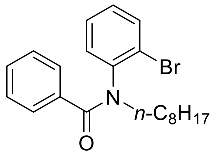

Isolated yield: 91%; ^{1}H NMR ($CDCl_3$): δ 7.52 (d, *J* = 7.6 Hz, 1H), 7.32 (d, *J* = 7.2 Hz, 2H), 7.10–7.23 (m, 4H), 7.00–7.08 (m, 2H), 4.18–4.26 (m, 1H), 3.40–3.47 (m, 1H), 1.63–

1.76 (m, 1H), 1.48–1.61 (m, 1H), 1.03–1.41 (m, 10H), 0.87 (t, $J = 6.8$ Hz, 3H); $^{13}C\{^1H\}$ NMR ($CDCl_3$): δ 170.6, 142.1, 136.3, 133.9, 132.0, 129.6, 129.0, 128.1, 128.0, 127.7, 123.5, 49.4, 31.9, 29.5, 29.4, 27.4, 27.2, 22.7, 14.2; IR (ATR): 2927, 2855, 1651, 757, 711 cm^{-1}; HRMS (DART+) m/z calcd. for $C_{21}H_{27}{}^{79}BrNO$, 388.1276 $[M+H]^+$; found, 388.1287.

5-Octyldibenzo[*b*,*f*][1,7]naphthyridin-6(5*H*)-one (2a)

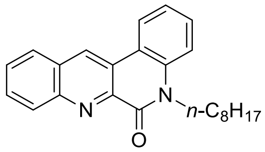

To a screw-capped test tube equipped with a magnetic stirring bar were added amide **1a** (44.1 mg, 0.100 mmol), potassium carbonate (42.0 mg, 0.304 mmol), tetrabutylammonium bromide (31.7 mg, 0.098 mmol), $Pd(OAc)_2$ (2.2 mg, 10 mol %) and triphenylphosphine (2.8 mg, 10 mol %). The mixture was dissolved in 3.1 mL of DMA and stirring was continued at 110 °C for 24 h. Water (3 mL) was added to the mixture after cooling to room temperature. The product was extracted with dichloromethane (2 mL) three times. The combined organic extracts were repeatedly washed with water (20 mL) and brine (20 mL). The organic layer was dried over anhydrous sodium sulfate and concentrated under reduced pressure to give a crude material, which was purified by silica gel column chromatography (hexane/MeOAc 1:1) to isolate 31.0 mg (87% yield) of **2a** as a colorless solid (NMR yield: 94%). Mp 85.1–86.6 ºC; ^1H NMR ($CDCl_3$): δ 8.98 (s, 1H), 8.43 (d, $J = 8.4$ Hz, 1H), 8.31 (dd, $J = 8.0$, 1.2 Hz, 1H), 7.95 (d, $J = 8.4$ Hz, 1H), 7.76 (ddd, $J = 8.0$, 7.6, 1.2 Hz, 1H), 7.62 (ddd, $J = 7.6$, 7.6, 1.2 Hz, 1H), 7.54 (ddd, $J =$ 8.4, 8.0, 1.2 Hz, 1H), 7.37 (d, $J = 8.4$ Hz, 1H), 7.30 (dd, $J = 8.0$, 7.6 Hz , 1H), 4.40 (dd, J = 8.0, 7.6 Hz, 2H), 1.76–1.88 (m, 2H), 1.44–1.56 (m, 2H), 1.18–1.42 (m, 8H), 0.86 (t, J = 6.8 Hz, 3H); $^{13}C\{^1H\}$ NMR ($CDCl_3$): δ 160.1, 148.4, 142.0, 136.7, 131.1, 130.5, 130.2, 130.1, 129.1, 128.7, 127.7, 126.3, 123.8, 122.7, 118.3, 115.4, 43.3, 31.9, 29.5, 29.3, 27.3, 27.1, 22.7, 14.2; IR (ATR): 2959, 2929, 2856, 1661, 751 cm^{-1}; HRMS (DART+) m/z calcd. for $C_{24}H_{27}N_2O$, 359.2123 $[M+H]^+$; found, 359.2134.

Synthesis of **2b**, **2c**, **8a**, and **8b** was carried out in a similar manner, spectroscopic characteristics and analytical properties were shown below.

6-Octyl-3-methylbenzo[*f*][1,7]naphthyridin-5(6*H*)-one (2b)

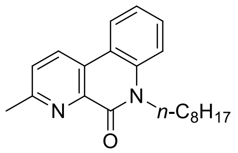

Isolated yield: 52%; ^{1}H NMR ($CDCl_3$): δ 8.49 (d, *J* = 8.4 Hz, 1H), 8.17 (dd, *J* = 7.6, 0.8 Hz, 1H), 7.53–7.58 (m, 1H), 7.53 (d, *J* = 8.4 Hz, 1H), 7.40 (d, *J* = 8.0 Hz, 1H), 7.30 (ddd, *J* = 7.6, 6.8, 0.8 Hz, 1H), 4.41 (dd, *J* = 7.6, 7.6 Hz, 2H), 2.79 (s, 3H), 1.80 (quint, *J* = 7.6 Hz, 2H), 1.49 (quint, *J* = 7.6 Hz, 2H), 1.19–1.40 (m, 8H), 0.86 (t, *J* = 6.8 Hz, 3H); ^{13}C{^{1}H} NMR ($CDCl_3$): δ 160.2, 160.1, 140.8, 136.9, 130.6, 130.0, 127.4, 127.1, 123.4, 122.5, 118.1, 115.3, 43.2, 31.9, 29.5, 29.3, 27.4, 27.1, 25.1, 22.7, 14.2; IR (ATR): 2954, 2926, 2854, 1663, 1598, 750 cm^{-1}; HRMS (DART+) m/z calcd. for $C_{21}H_{27}N_2O$, 323.2123 $[M+H]^+$; found, 323.2117.

6-Octylbenzo[*f*][1,7]naphthyridin-5(6*H*)-one (2c)

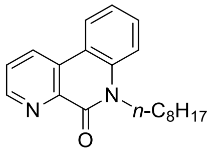

Isolated yield: 62%; ^{1}H NMR ($CDCl_3$): δ 8.96 (dd, *J* = 4.4, 1.2 Hz, 1H), 8.60 (d, *J* = 8.4 Hz, 1H), 8.22 (d, *J* = 8.0 Hz, 1H), 7.66 (dd, *J* = 8.4, 4.4 Hz, 1H), 7.57 (ddd, *J* = 8.0, 7.2, 0.8 Hz, 1H), 7.41 (d, *J* = 8.0 Hz, 1H), 7.32 (ddd, *J* = 7.6, 7.2, 0.8 Hz, 1H), 4.42 (dd, *J* = 8.0, 7.2 Hz, 2H), 1.80 (quint, *J* = 7.6 Hz, 2H), 1.49 (quint, *J* = 7.6 Hz, 2H), 1.19–1.40(m, 8H), 0.86 (t, *J* = 6.8 Hz, 3H); ^{13}C{^{1}H} NMR ($CDCl_3$): δ 160.1, 150.7, 141.5, 137.2, 130.6, 130.4, 129.8, 126.6, 123.7, 122.6, 117.8, 115.4, 43.2, 31.9, 29.5, 29.3, 27.4, 27.1, 22.7, 14.2; IR (ATR): 2956, 2927, 2854, 1662, 1608, 750 cm^{-1}; HRMS (DART+) m/z calcd. for $C_{20}H_{25}N_2O$, 309.1967 $[M+H]^+$; found, 309.1974.

5,9-Diethylbenzo[*f*]quino[3,4-*b*][1,7]naphthyridine-6,8(5*H*,9*H*)-dione (4)

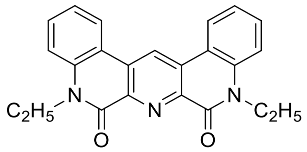

NMR yield: 87%; spectroscopic characteristics and analytical properties of **4** were identical with those of authentic sample[2]

2-Dihydro-2-octylquinolino[3,4-*b*][1,10]phenanthroline (6a)

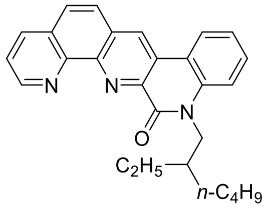

Isolated yield: 51%; spectroscopic characteristics and analytical properties of **6a** were identical with those of authentic sample[1].

5,12-Dioctyl-5,12-dihydroquinolino[4,3-*j*]phenanthridin-6,13-dione (8a)

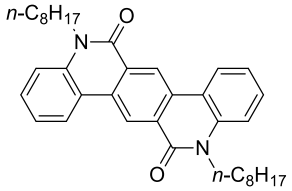

NMR yield: 81%; Mp 170.3–171.9 ºC; ^{1}H NMR ($CDCl_3$): δ 9.48 (s, 2H), 8.54 (dd, *J* = 8.0, 1.2 Hz, 2H), 7.59 (ddd, *J* = 8.0, 7.6, 1.2 Hz, 2H), 7.44 (d, *J* = 8.0 Hz, 2H), 7.38 (dd, *J* = 8.0, 7.6 Hz, 2H), 4.43 (dd, *J* = 8.0, 7.6 Hz, 4H), 1.85 (quint, *J* = 8.0 Hz, 4H), 1.54 (quint, *J* = 8.0 Hz, 4H), 1.21–1.47 (m, 16H), 0.89 (t, *J* = 6.8 Hz, 6H); ^{13}C{^{1}H} NMR ($CDCl_3$): δ 161.0, 137.1, 132.3, 130.0, 128.1, 124.3, 123.0, 122.9, 119.4, 115.3, 43.2, 31.9, 29.5, 29.4, 27.5, 27.3, 22.8, 14.2; IR (ATR): 2954, 2926, 2853, 1648, 789 cm^{-1}; HRMS (DART+) m/z calcd. for $C_{36}H_{45}N_2O_2$, 537.3481 [M+H]$^+$; found, 537.3499.

5-Octyl-6(5*H*)-phenanthridinone (8b)

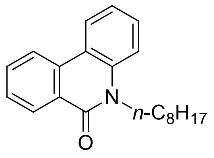

NMR yield: 89%; ^{1}H NMR ($CDCl_3$): δ 8.56 (dd, *J* = 8.0, 1.2 Hz, 1H), 8.29 (ddd, *J* = 8.8, 8.0, 1.2 Hz, 2H), 7.76 (ddd, *J* = 7.6, 7.6, 1.2 Hz, 1H), 7.59 (ddd, *J* = 7.6, 6.8, 0.8 Hz, 1H), 7.55 (ddd, *J* = 7.6, 6.8, 1.2 Hz, 2H), 7.41 (d, *J* = 8.0 Hz, 1H), 7.31 (ddd, *J* = 7.6, 7.6, 0.8 Hz, 1H), 4.38 (dd, *J* = 8.0, 7.6 Hz, 2H), 1.80 (quint, *J* = 8.0 Hz, 2H), 1.50 (quint, *J* = 8.0 Hz, 2H), 1.20–1.43 (m, 10 H), 0.88 (t, *J* = 6.8 Hz, 3H); ^{13}C{^{1}H} NMR ($CDCl_3$): δ 161.3, 137.1, 133.5, 132.3, 129.5, 128.8, 127.9, 125.5, 123.4, 122.2, 121.5, 119.4, 115.1, 42.8, 31.7, 29.3, 29.2, 27.4, 27.1, 22.6, 14.0; IR (ATR): 2954, 2927, 2854, 1650, 800, 748 cm^{-1}; HRMS (DART+) m/z calcd. for $C_{21}H_{26}NO$, 308.2014 [M+H]$^+$; found, 308.2008.

***N*-(2-Bromophenyl)-*N*-octyl-2-quinolinecarboxamide (1a)**

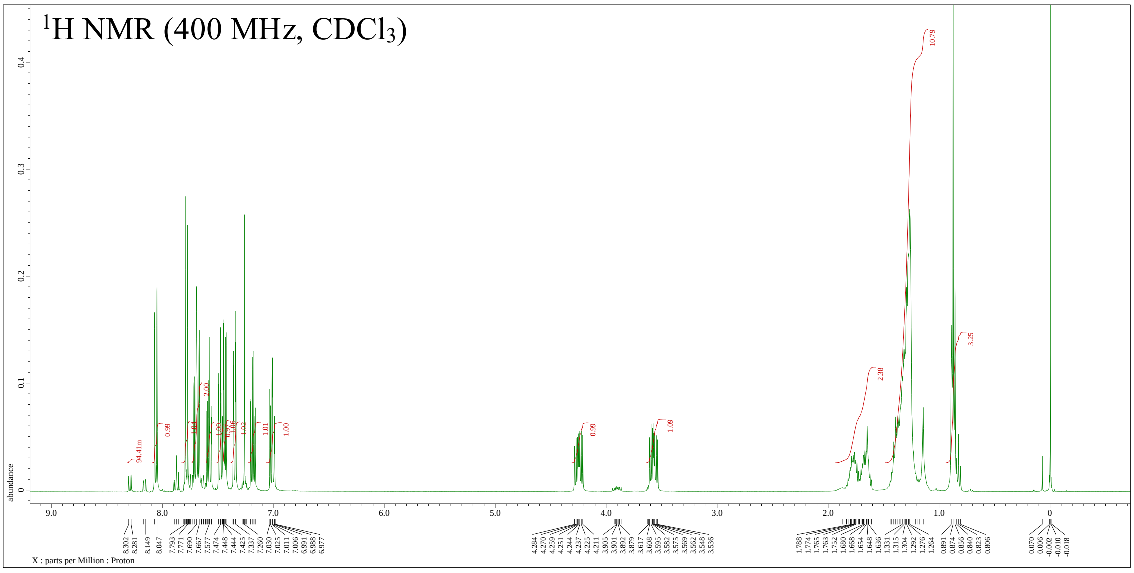

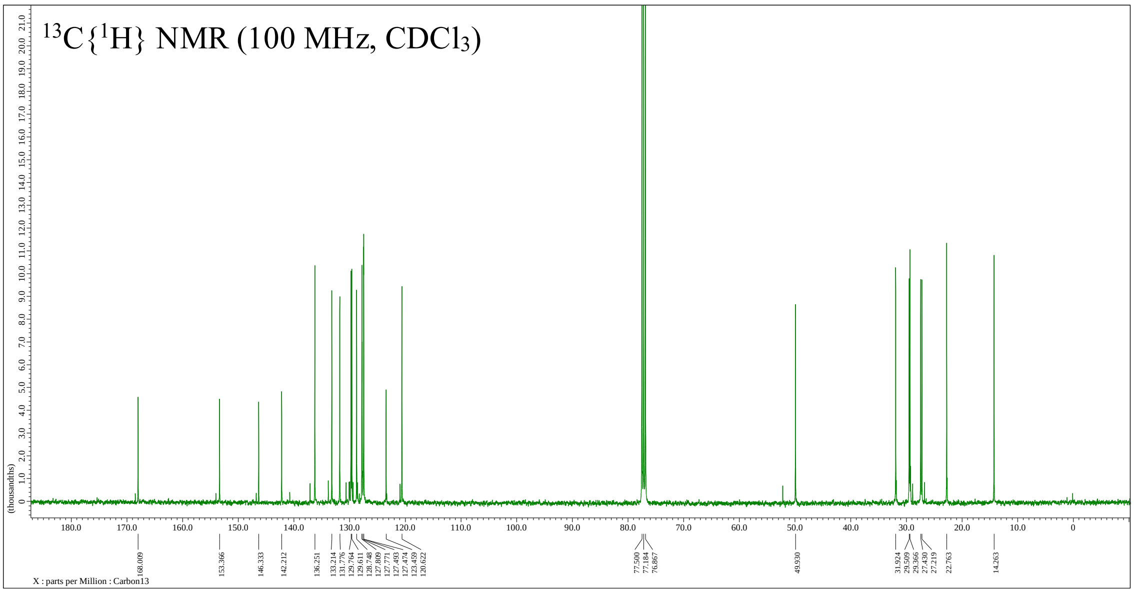

Peak list contains amide-derived rotational isomer signals.

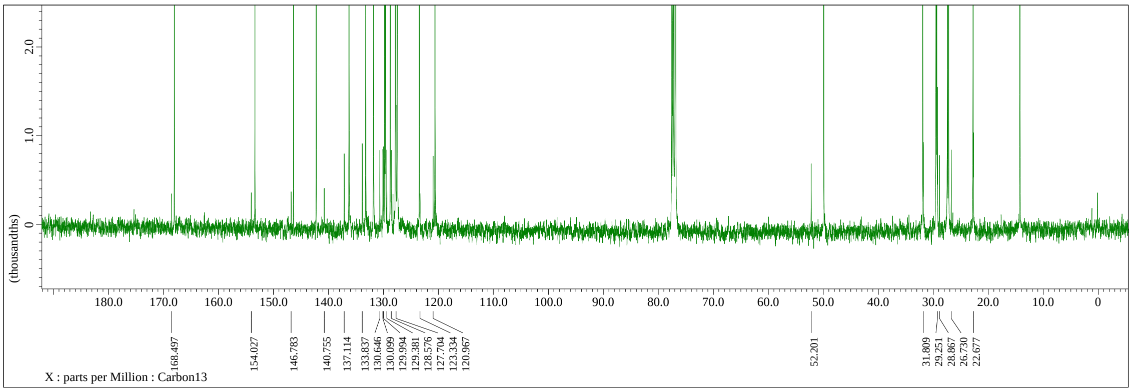

***N*-(2-Bromophenyl)-*N*-octyl-6-methylpicolinamide (1b)**

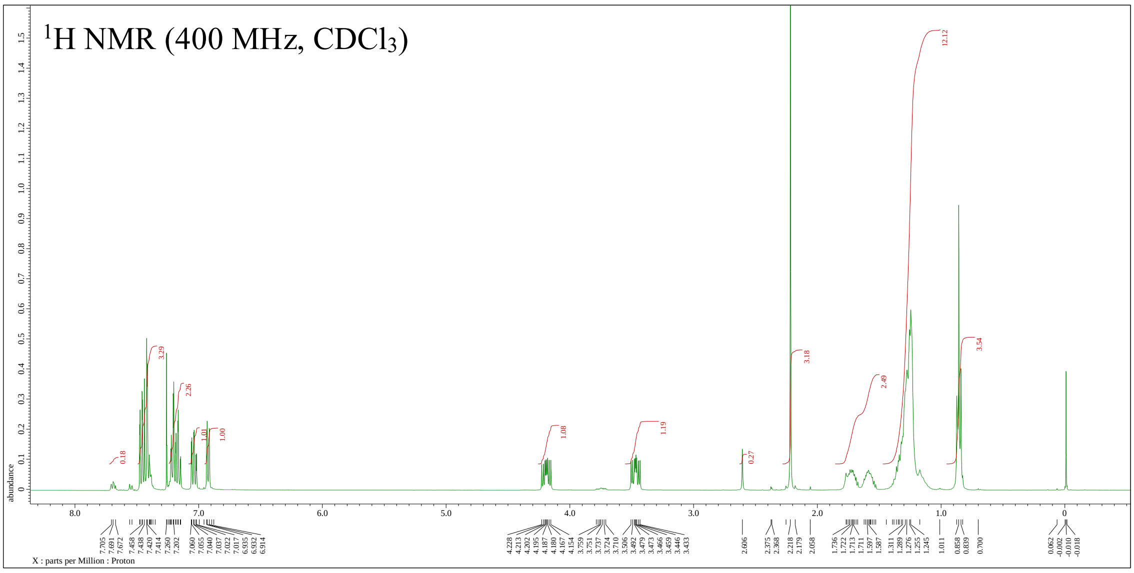

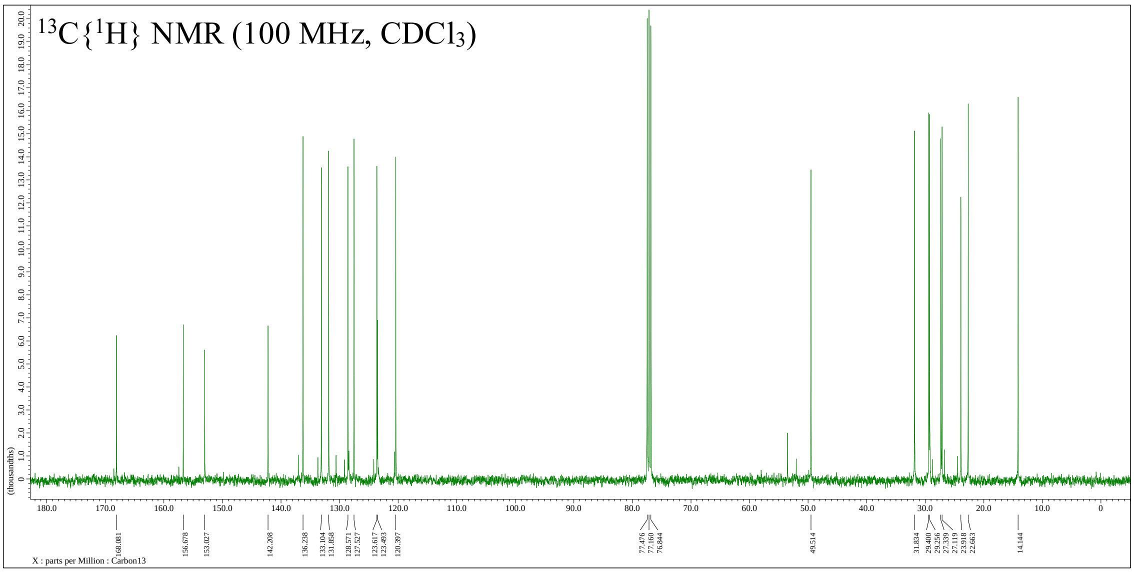

Peak list contains amide-derived rotational isomer signals.

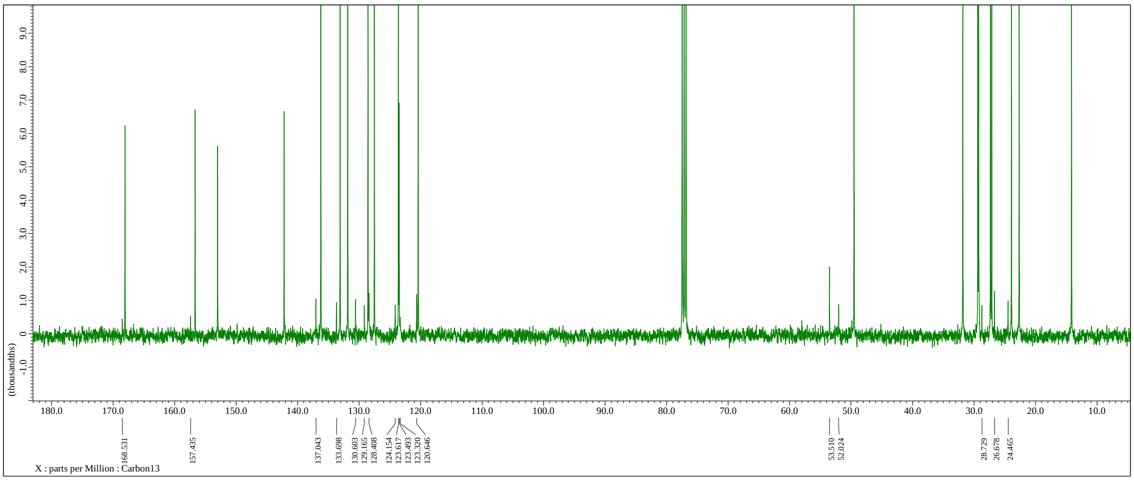

N-(2-Bromophenyl)-*N*-octyl-picolinamide (1c)

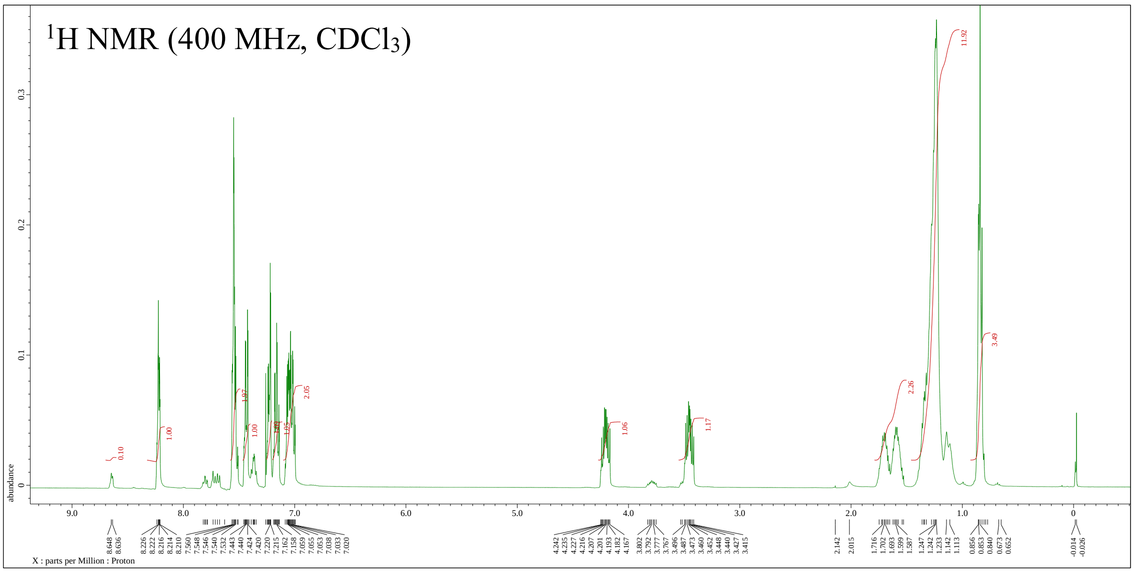

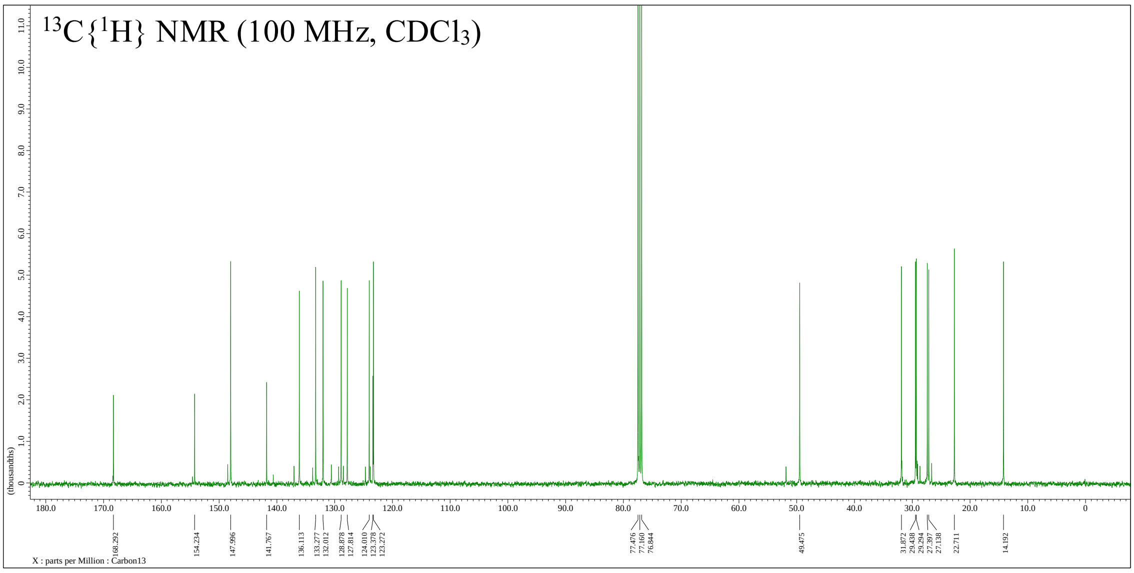

Peak list contains amide-derived rotational isomer signals.

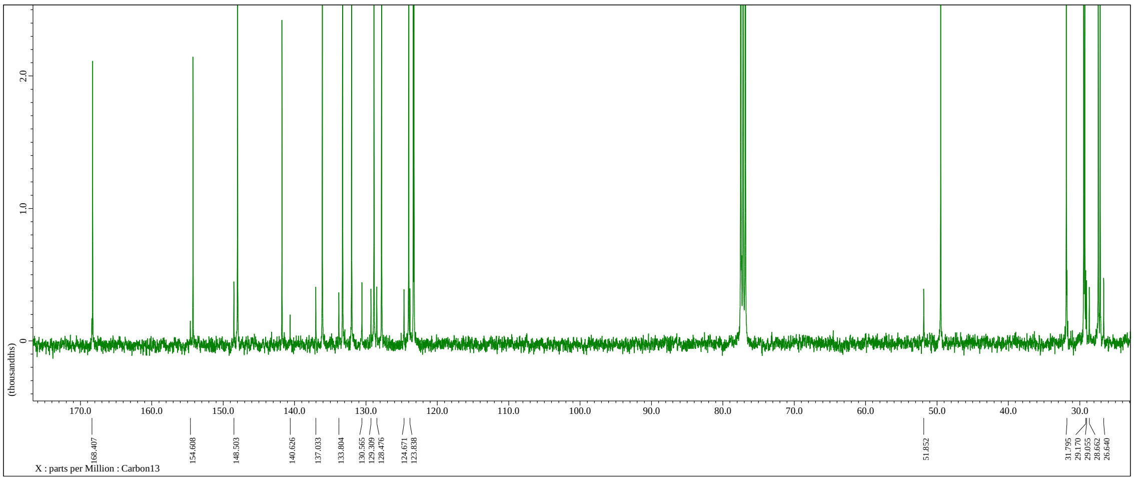

N^1,N^4-Bis(2-bromophenyl)-N^1,N^4-dioctylterephthalamide (7a)

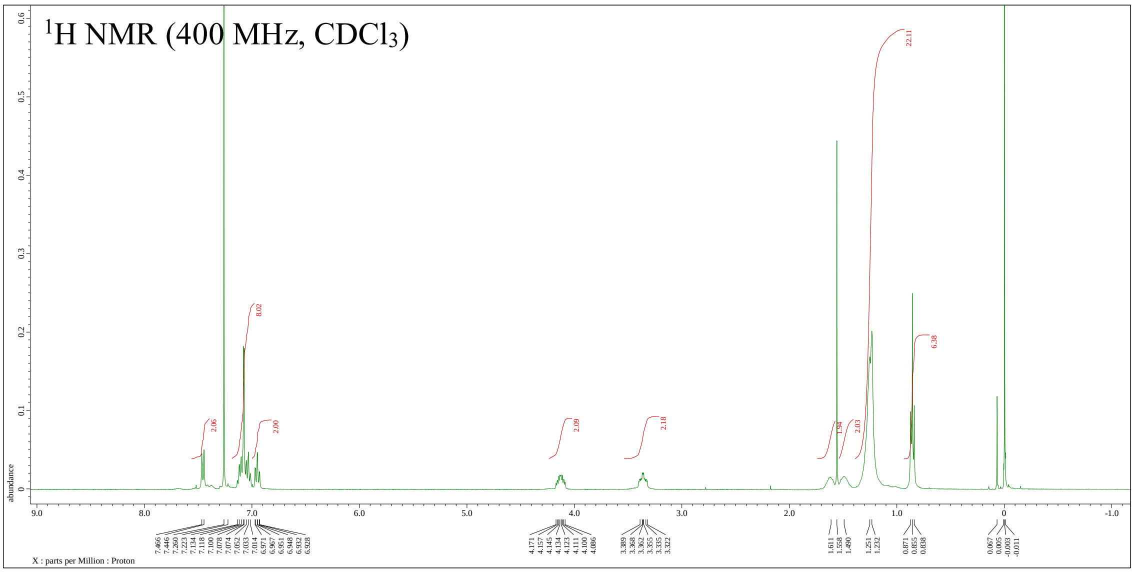

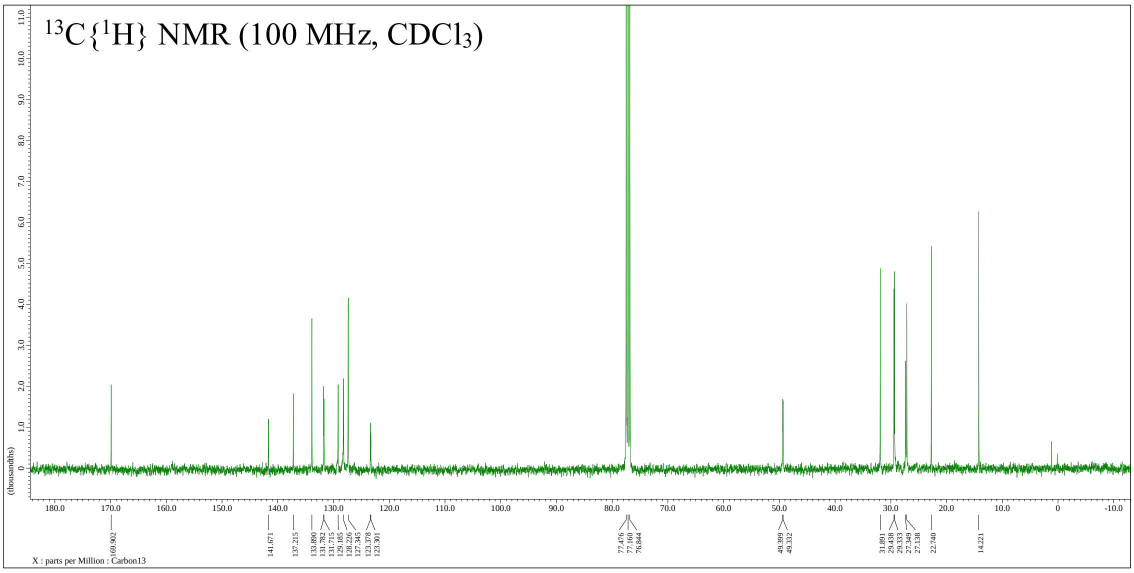

N-(2-Bromophenyl)-*N*-octylbenzamide (7b)

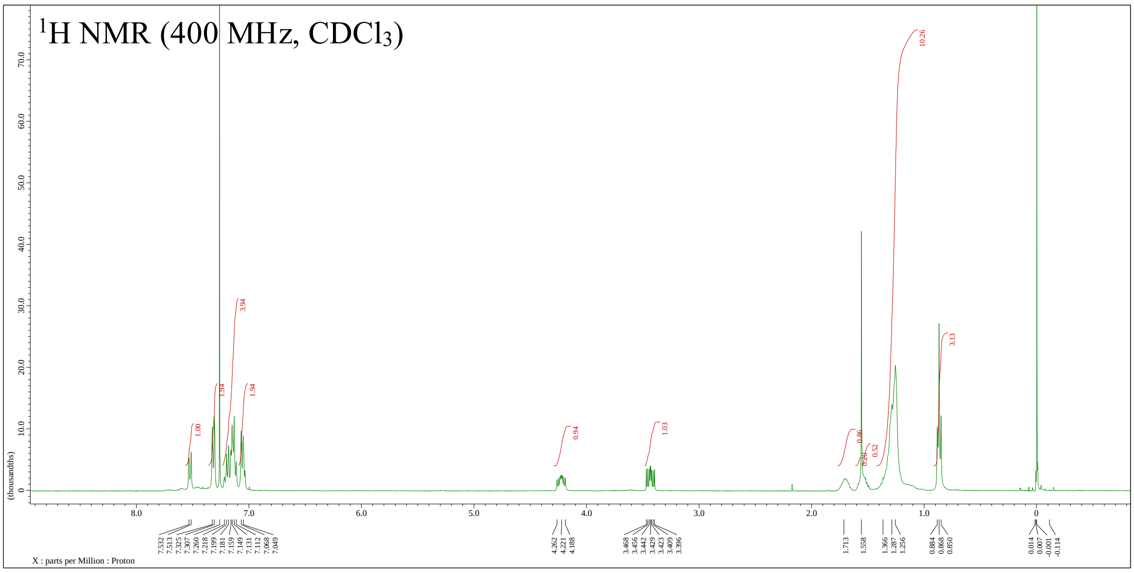

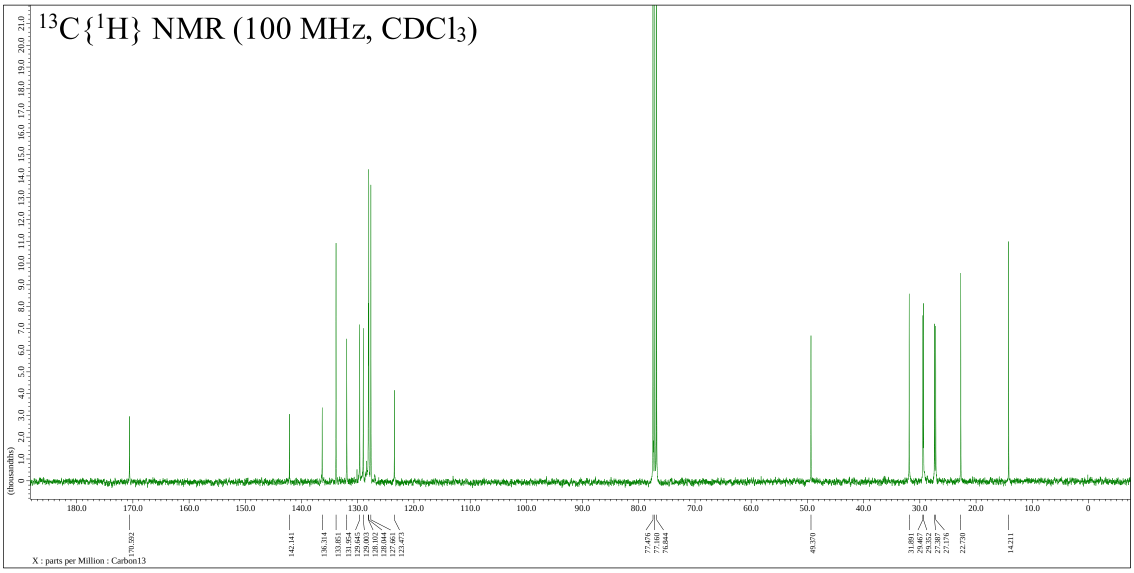

5-Octyldibenzo[*b*,*f*][1,7]naphthyridin-6(5*H*)-one (2a)

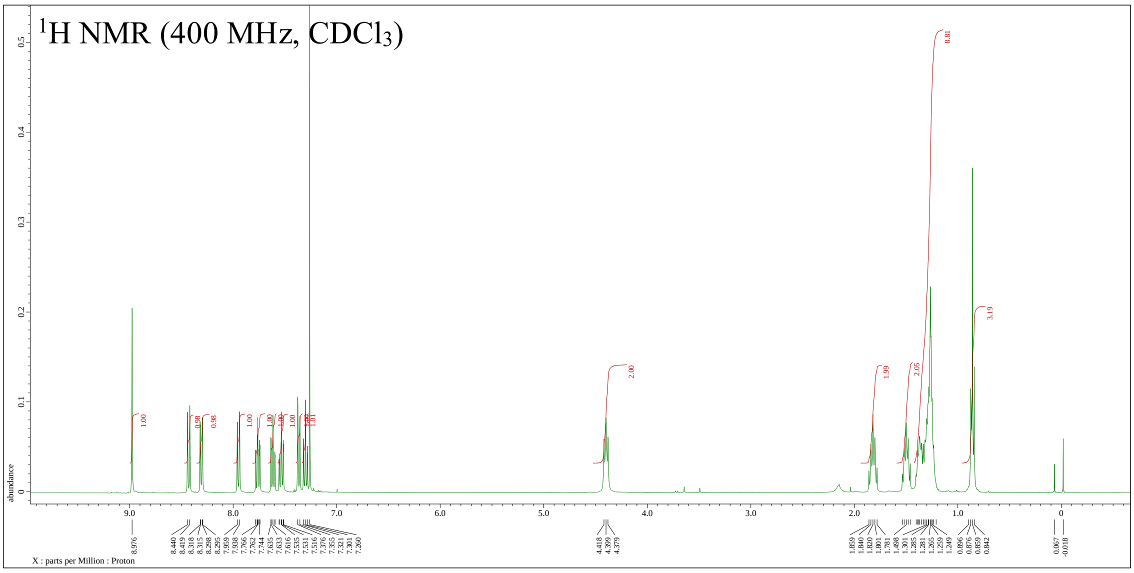

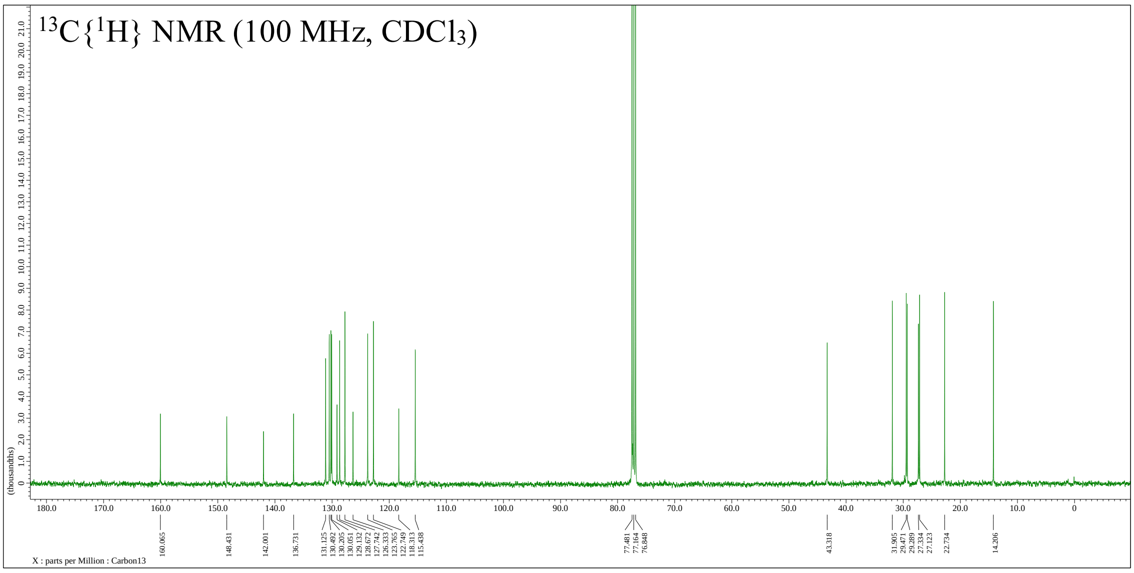

6-Octyl-3-methylbenzo[*f*][1,7]naphthyridin-5(6*H*)-one (2b)

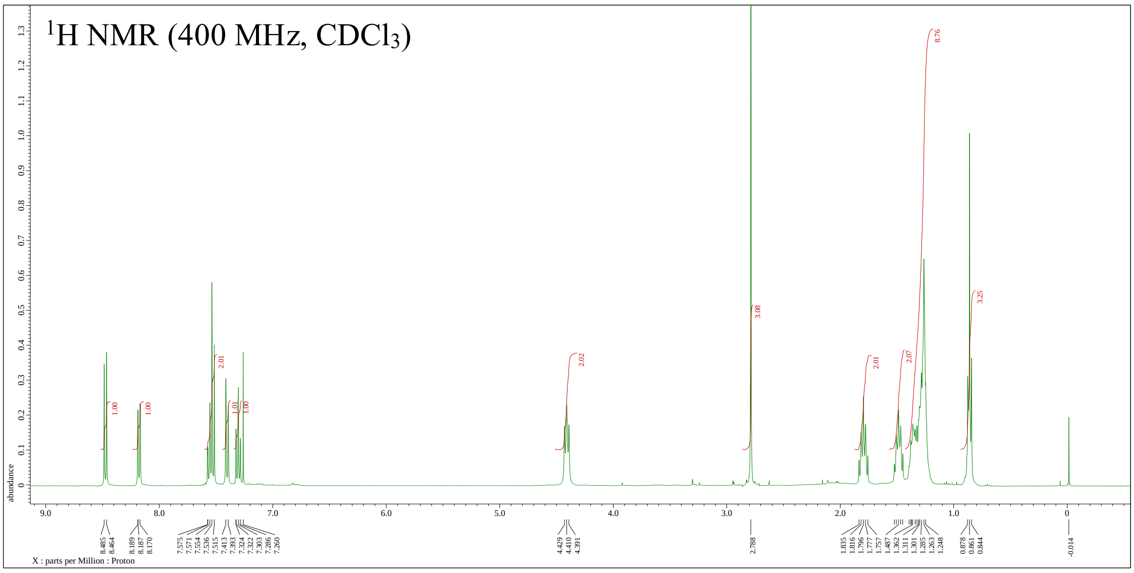

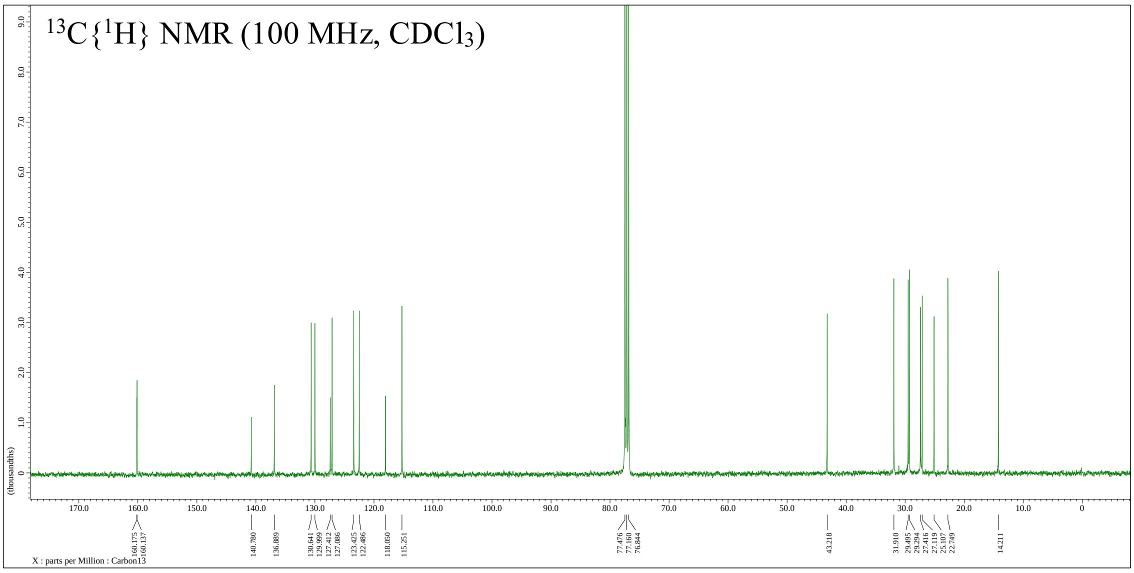

6-Octylbenzo[*f*][1,7]naphthyridin-5(6*H*)-one (2c)

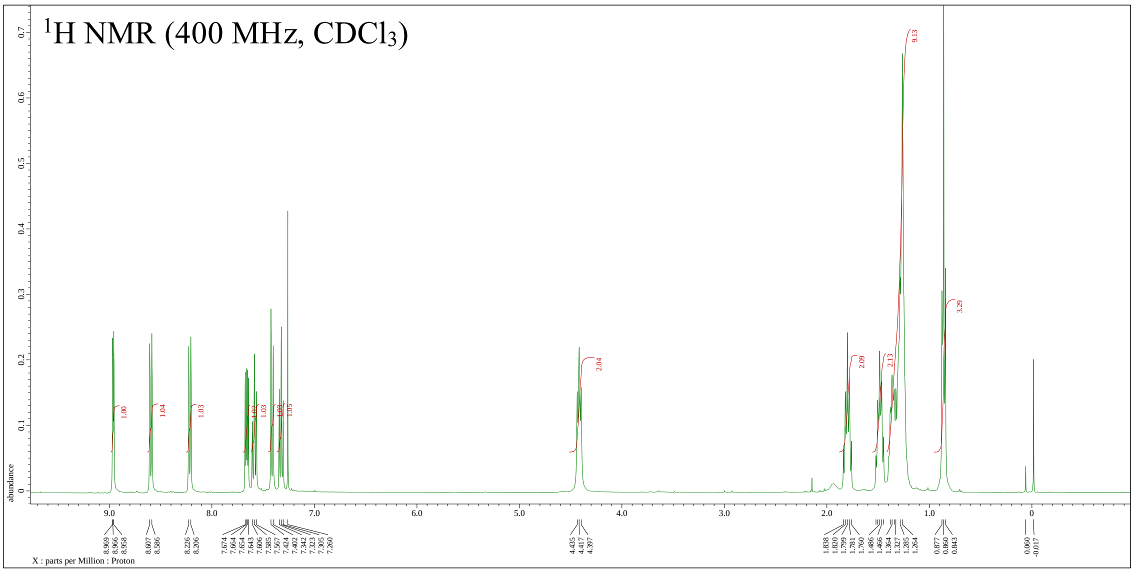

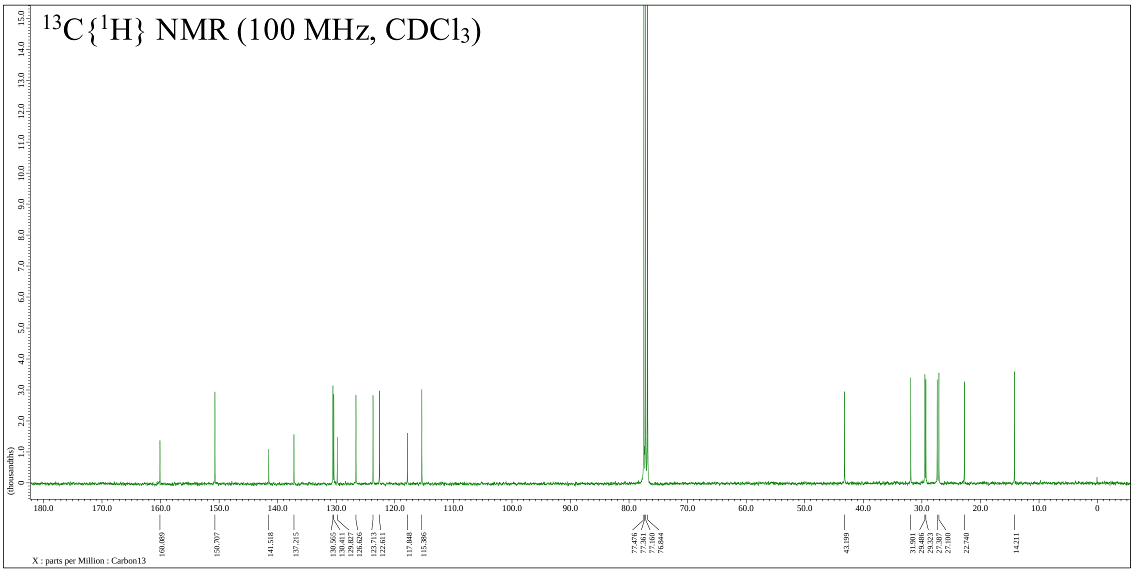

5,12-Dioctyl-5,12-dihydroquinolino[4,3-*j*]phenanthridin-6,13-dione (8a)

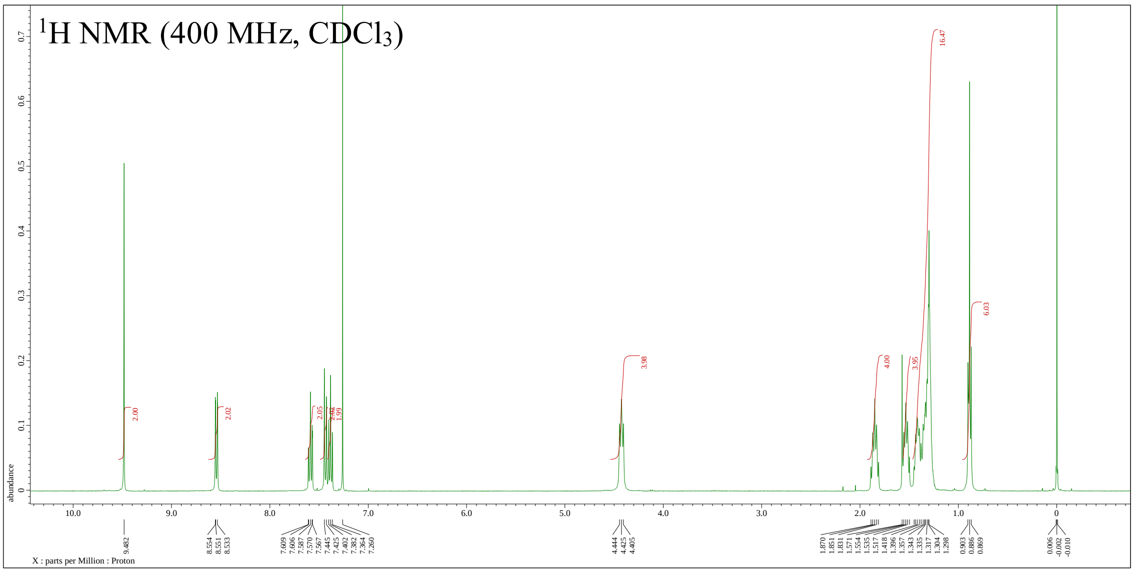

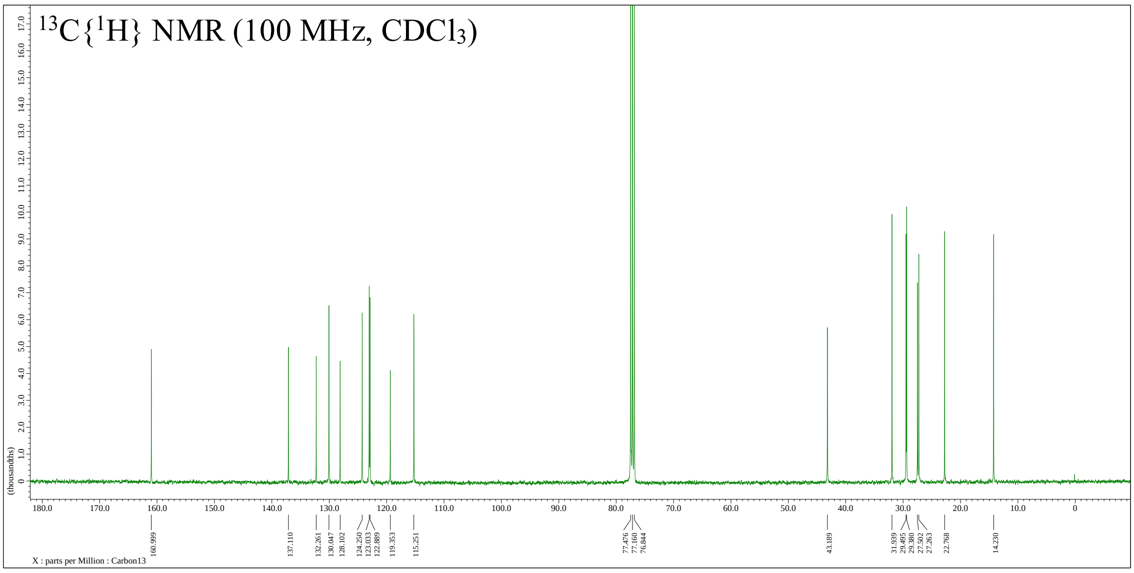

5-Octyl-6(5*H*)-phenanthridinone (8b)

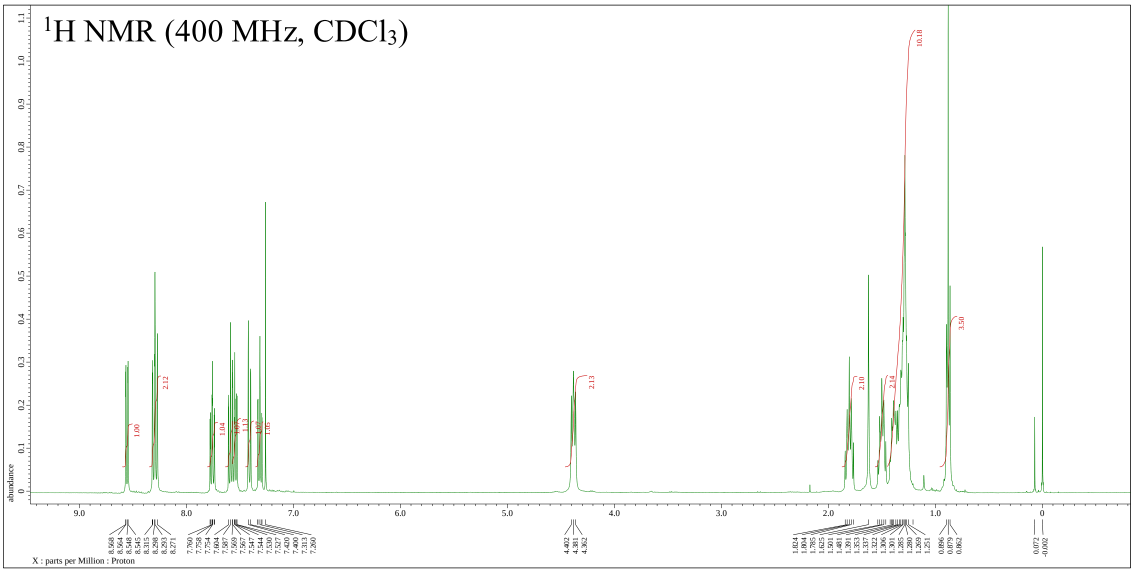

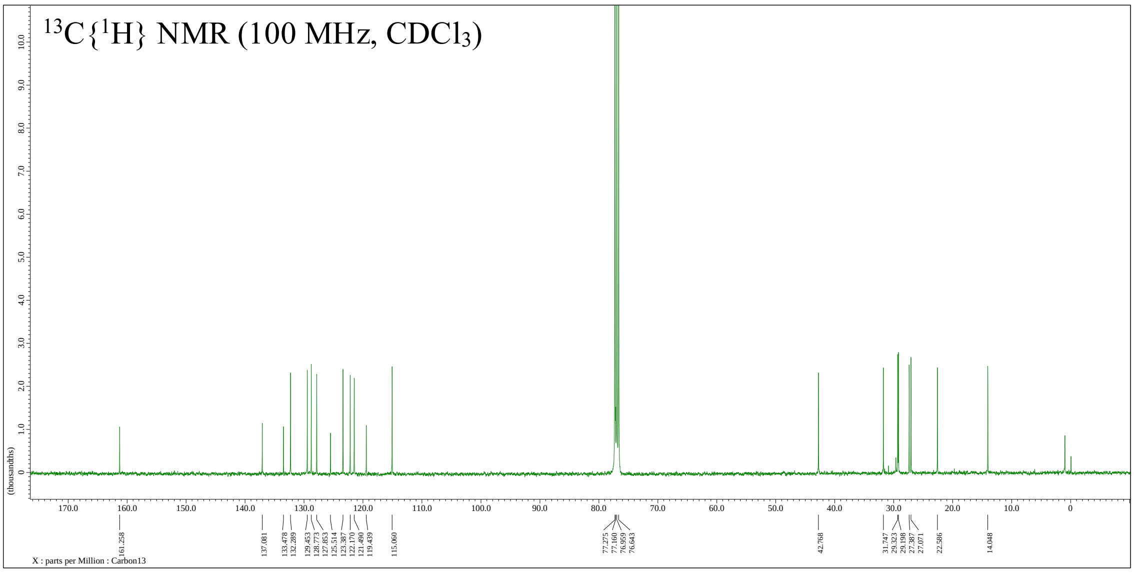

Reference

(1) Asano, T.; Nakanishi, Y.; Sugita, S.; Okano, K.; Narita, H.; Kobayashi, T.; Yaita, T.; Mori, A. *ChemRxiv* **2024**. This content is a preprint and has not been peer-reviewed. doi:10.26434/CHEMRXIV-2024-8LPMZ

(2) Majumdar, K. C.; De, N.; Chakravorty, S. *Synth. Commun.* **2010**, *41*, 121–130. doi:10.1080/00397910903531870

(3) Alyapyshev, M.; Ashina, J.; Dar’In, D.; Kenf, E.; Kirsanov, D.; Tkachenko, L.; Legin, A.; Starova, G.; Babain, V. *RSC Adv.* **2016**, *6*, 68642–68652. doi:10.1039/c6ra08946a
